# Anxiety due to Dental Treatment and Procedures among University Students and Its Correlation with Their Gender and Field of Study

**DOI:** 10.1155/2013/647436

**Published:** 2013-03-14

**Authors:** Mohd G. Sghaireen, Abdalwhab M. A. Zwiri, Ibrahim A. Alzoubi, Sadeq M. Qodceih, Mahmoud K. AL-Omiri

**Affiliations:** ^1^Department of Prosthodontics, Faculty of Dentistry, Al-Jouf University, Sakaka 42421, Saudi Arabia; ^2^Department of Periodontics and Oral Medicine, Faculty of Dentistry, Al-Jouf University, Sakaka 42421, Saudi Arabia; ^3^Department of Orthodontics, Faculty of Dentistry, Al-Jouf University, Sakaka 42421, Saudi Arabia; ^4^Department of Prosthodontics, Faculty of Dentistry, University of Jordan, Amman 11942, Jordan

## Abstract

*Aim of Study*. To investigate dental anxiety levels among university students and its relation with their specialty and gender. *Materials and Methods*. 850 undergraduate university students were recruited into the study. The Modified Corah Dental Anxiety Scale (MDAS) was used to measure the levels of their dental anxiety. 700 questionnaires were returned, 390 females and 310 males (response rate of 0.92% among females, 0.73% among males). The MDAS score ranged from 5 to 25. Patients were considered to suffer from high dental anxiety if they scored 13 to 20 points. Statistical analysis significance was set at *P* ≤ 0.05. *Results*. Seven hundred students participated in this study including 13% of medical students, 10% of dental students, 58% of arts students, and 18% of computer science students. Medical and dental students were less anxious than arts and computer science students (*P* < 0.05). Local anesthesia injection was the most fearful dental procedure (*P* < 0.05). Females were more anxious than males (*P* < 0.05). *Conclusion*. Male students were less anxious than female students. Students from medical background faculties were less anxious than students from nonmedical faculties. Lack of adequate dental health education may result in a higher level of dental anxiety among nonmedical students in Saudi Arabia.

## 1. Introduction

Dental anxiety is a frequent problem among dental patients. It is a multisystem reaction to a perceived threat or danger [1, 2]. It reflects a combination of biochemical alterations in the body and patient's personal history, memory, and social state. The presence of dental anxiety is not a dilemma for patients only but also for the dental professionals themselves; and sometimes it renders the treatment more complicated to be accomplished successfully [3, 4].

Oral diseases are chief public health concerns, and their prevalence could be boosted by dental anxiety [5]. In addition, dental anxiety might influence patient-dentist relationship, obscure proper diagnosis of the genuine dental problem, and result in deterioration of patients' personal oral health [6, 7]. Kirova et al. found that it is the 25-26-year-olds that tend to experience higher dental anxiety than other people [8]. This could be attributed to the diverse impacts of a number of psychological factors in this age range that can induce dental fear and anxiety [9, 10].

Several studies reported significantly higher levels of dental anxiety among females; however, the clinical significance of this gender difference has been questioned [3, 11]. Taani found that dental anxiety was lower among Jordanian private school children than those who are attending public school [4]. However, dental anxiety was found among the most common reasons that trigger irregular attendance in two-thirds of public school children and half of private school students [4]. 

Some researchers suggested that dental students have lower levels of dental anxiety in comparison with students in other majors which might be due to lack of adequate dental health education that results in a high level of dental anxiety among nondental university students [10]. The dental literature lacks sufficient information about correlation between students ‘gender, different fields of study' and the levels of dental anxiety. 

No previous studies regarding this topic are present among Saudi undergraduate university students; this provoked the conduction of this study.

The aim of the present study was to investigate the levels of dental anxiety among undergraduate students in Al-Jouf University, Saudi Arabia. In addition, the study explored the sources of dental anxiety, and the impact of gender and field of study on the perceived dental anxiety.

## 2. Materials and Methods

Seven hundred and fifty-five undergraduate university students (390 females and 310 males) were recruited into this study from the Faculties of Medicine, Dentistry, Engineering, and literature in Al-Jouf University, Sakaka, Saudi Arabia. Participants' age ranged from 17 to 28 years old (mean age = 21.34 ± 1.8 years old).

This study was approved by the research committee at the Faculty of Dentistry, Al-Jouf University. 

To be included in the study, participants should have no medical disease (including mental problems) that might affect their ability to understand and/or score the questionnaires. 

Each participant was given a brief explanation of the study, and an informed consent was obtained from each participant before being recruited into the study.

An invitation to participate in the study was directly extended to 800 undergraduate students. However, seven hundred students (390 females and 310 males) responded and accepted to participate in the study. This accounts for a response rate of 97.5% among females and 77.5% among males.

The Modified Corah Dental Anxiety Scale (MDAS) [11] was used to measure the levels of dental anxiety among students. The questionnaire consists of five questions that test participants' anxiety due to certain dental procedures and situations. These include the following.

1. If you were going to your dentist for treatment tomorrow, how would you feel?
2. If you were sitting in the waiting room, how would you feel?
3. If you were about to have a tooth drilled, how would you feel?
4. If you were about to have your teeth scaled and polished, how would you feel?
5. If you were about to have a local anesthetic injection in your gum, how would you feel?

A simplified 5-point-scale answering scheme was devised for each question ranging from not anxious to extremely anxious and measured in points from 1 to 5, respectively. 

In order to obtain the total score of the scale, the scores for each of the 5-item responses were summed. The maximum possible score of the scale is 25, and the minimum score is 5. 

The final assessment of the level of anxiety is given by the sum of points of scale items: less than score 8 is considered to be no anxiety, 9 to 12 is moderate anxiety, 13 to 14 is high anxiety, and 15 to 20 is severe anxiety bordering on phobia. Patients were considered to suffer from high dental anxiety if they scored 13 to 20 points [8, 9, 12]. 

This scale is considered to be valid, reliable, brief, accessible, and easy to use; thus, it was used to assess the levels of dental anxiety in this study [13].

The Modified Corah Dental Anxiety Scale was administered to the participants, and the process of completing the questionnaire was supervised by the investigator. The participants were offered assistance if they faced any problem during scoring the questionnaire.

*Statistical Analysis. *The data were analyzed using the SPSS computer software (Statistical Package for the Social Sciences, version 19.0, SPSS Inc., Chicago, IL, USA). Simple frequency tables were generated, and then the ANOVA and Post Hoc tests were used to identify the differences between genders as well as between different study fields. For all statistical analysis, the significance level was set at *P* ≤ 0.05.

## 3. Results

In total, 700 completed questionnaires were returned from participants. The study fields of the participants included Medicine (13% of participants), dentistry (10%), Arts (58%), and Computer science (18%).  Table 1 presents the distribution of the study sample by gender and the mean score of the Modified Corah Dental Anxiety scale. Anxiety levels were found to be higher among females (*P* < 0.05).

Table 2 presents the distribution of the study sample by the study field and the total scores of the MDAS. Dental students reported the minimum anxiety score (mean = 12.26 ± 4.852), whereas art students scored the highest levels of anxiety (mean = 14.04 ± 4.950). 

Levels of anxiety were not significantly different between dental and medical students (*P* > 0.05) (Table 3). On the other hand, dental and medical students had less anxiety levels than arts and computer science students (*P* < .05) (Table 3). 

The study level (year of study) of nondental students had no relationship to the levels of dental anxiety (*P* > 0.05). However, first-year dental students had higher levels of dental anxiety than senior ones (*P* > 0.05). 

The most anxious situation of all dental procedures was the injection of local anesthesia followed by drilling of teeth. Nearly 36% of the participants (250 participants) were extremely anxious about having a local anesthetic injection, while 27% (186 participants) were extremely anxious about tooth drilling (Table 4).

## 4. Discussion

Despite the technological advances made in modern dentistry, anxiety about dental treatment and fear of pain associated with it remain prevalent [14]. Most of the participants in this study were anxious, 76.8% (with high MDAS score = 13–20). This was consistent with the results of other studies which found a considerably greater percentage of participants with dental anxiety [10, 15–17]. 

However, other studies found that the most tested participants were anxiety-free [8]. The Modified Dental Anxiety Scale is considered to be valid, reliable, brief, accessible, and easy to use; thus, it was used to assess the levels of dental anxiety in this study [13].

High anxiety level among the students in Saudi Arabia might result from lack of dental health education which in turn might end with poor compliance and attitude, or it might be linked to personality characteristics, fear of pain, past traumatic dental experiences in childhood, and dentally anxious family members or peers. High anxiety level would make it more difficult to manipulate patients and yield difficult patients and thus increase the levels of dental profession-related stress [13, 18].

In the present study, dental and medical students were less anxious than arts and computer science students. This can be explained by the increased awareness, education, and professional development and acquired clinical experience of dental and medical students. These findings are consistent with the conclusions of other researchers that dental anxiety is less prevalent in dental students [10]. 

Also, first-year students showed much greater anxiety score than senior students. These findings concur the results reported by other researchers who claimed that dental anxiety declined in advanced years of study [10, 19, 20].

In relation to gender, it was found that dental anxiety was greater among females. This can be explained by the fact that males are more emotionally stable than females [10, 21–23]. These findings corroborated the results from previous studies that showed higher levels of anxiety among females [10, 21–23]. Other studies showed no difference between the two genders [9, 24–26]. These differences among researchers might be due to cultural differences.

Local anesthesia injection was the most fearful situation of all dental procedures, followed by drilling of the teeth. 35.7% of the students were extremely anxious if they were going to have local anesthesia injection. Meanwhile, 26.6% of the whole students were extremely anxious about drilling their teeth. Needle phobia is age related but should be considered as a separate phenomenon [27]. It is not specific for dental anxiety and is related to other painful treatments [28]. 

The above results were in agreement with previous studies [29–31]. 

Oral diseases are important public health concerns, and their prevalence is increased by dental anxiety, and affects quality of life. These results underscore the need for population-based studies to identify the correlates of dental anxiety for better dental health in Saudi Arabia. Also, it is recommended to amend university curriculum to include adequate dental education for different specialties. Moreover, implication of oral health education at school level will be very helpful in this regard.

## 5. Conclusions

1. Dental and medical students had lower levels of anxiety than arts and computer science students. Also, first-year dental students had significantly higher levels of dental anxiety than senior students. Increased awareness, education, professional development and acquired clinical experience of dental and medical students might be the reason.
2. Females demonstrated higher anxiety levels than men.
3. Local anesthesia and drilling of teeth were major sources of dental anxiety.

## Figures and Tables

**Table 1:** The distribution of the study sample by gender and the mean score of the Modified Corah Dental Anxiety scale.

Gender	*N*	Mean score	Standard deviation	Standard error	95% Confidence interval for mean		Significance*
					Lower bound	Upper bound
Male	310	12.69	5.242	0.298	12.10	13.27	0.000
Female	390	14.50	4.489	0.227	14.05	14.94	

Total	700	13.70	4.916	0.186	13.33	14.06	

*Significance using ANOVA test (*P* ≤ 0.05).

**Table 2:** Distribution of the study sample by the field of study and the mean total anxiety score (*n* = 700).

Field of study	Number (%)	Mean total anxiety score (standard deviation)
Dental student	72 (10.3)	12.26 (4.852)
Medical student	90 (12.9)	12.79 (5.044)
Arts student	411 (58.7)	14.04 (4.950)
Computer science student	127 (18.1)	14.02 (4.573)

Total	700 (100)	13.7 (4.916)

**Table 3:** Post Hoc test of the variance in mean anxiety scores between dental students and other study fields.

Groups	Standard error	Mean difference	Significance (*P* ≤ 0.05)	95% Confidence interval for mean	
				Lower bound	Upper bound
Dentistry versus medicine	0.775	−0.536	0.489	−2.06	0.99
Dentistry versus arts	0.632	−1.784	0.005	−3.02	−0.54
Dentistry versus computer science	0.727	−1.766	0.015	−3.19	−0.34

**Table 4:** The distribution of participants according responses to the items of Modified Dental Anxiety Scale (*n* = 700).

Item	Not anxious	Slightly anxious	Fairly anxious	Anxious	Extremely anxious
If you went to your dentist for treatment tomorrow, how would you feel?	290 (41.4%)	225 (32.1%)	70 (10%)	75 (10.7%)	40 (5.7%)
If you were sitting in the waiting room, how would you feel?	247 (35.3%)	206 (29.4%)	117 (16.7%)	76 (10.9%)	54 (7.7%)
If you were about to have a tooth drilled, how would you feel?	79 (11.3%)	132 (18.9%)	133 (19.0%)	170 (24.3%)	186 (26.6%)
If you were about to have your teeth scaled and polished, how would you feel?	228 (32.6%)	184 (26.3%)	114 (16.3%)	102 (14.6%)	72 (10.3%)
If you were about to have a local anesthetic injection in your gum, how would you feel?	67 (9.6%)	114 (16.3%)	123 (17.6%)	145 (20.7%)	250 (35.7%)
